# Exosomes in Metabolic Bone Diseases: Regulators, Biomarkers and Targeted Delivery Systems

**DOI:** 10.3390/biomedicines14092092

**Published:** 2026-09-17

**Authors:** Linxiao Wang, Xi Gao, Shasha Jiang, Yiran Zhang, Jiangang Xie, Haifan Yang, Yinghui Li, Lin Liu

**Affiliations:** 1 Department of Clinical Laboratory, Honghui Hospital, Xi’an Jiaotong University, Xi’an 710054, China; 2 Department of Critical Care Medicine, Honghui Hospital, Xi’an Jiaotong University, Xi’an 710054, China; 3 Department of Emergency, Honghui Hospital, Xi’an Jiaotong University, Xi’an 710054, China; 4 Department of Anesthesiology, The Second Affiliated Hospital of Xi’an Medical University, Xi’an 710021, China

**Keywords:** exosomes, metabolic bone diseases, bone homeostasis, extracellular vesicles, targeted delivery, non-coding RNA biomarkers, engineered modification

## Abstract

**Background:** Metabolic bone diseases represent prevalent global disorders characterized by disrupted bone homeostasis and chronic inflammation. This review utilizes a broad working definition encompassing osteoporosis and other classic metabolic bone disorders, including osteoarthritis, a primarily degenerative joint disease marked by significant metabolic-driven subchondral bone remodeling. Conventional diagnostic modalities prove inadequate for early-stage disease screening, while classical pharmacological interventions face limitations due to unsatisfactory targeting performance and severe long-term adverse reactions. An urgent clinical need persists for innovative diagnostic biomarkers and targeted therapeutic strategies. Exosomes, a sub-population of small extracellular vesicles (sEVs), are natural nanoscale intercellular vesicles derived from multivesicular bodies, playing critical regulatory roles in bone metabolism and offering substantial clinical translational potential. This review systematically elaborates the regulatory networks associated with exosomes, the biomarker potential of exosomal non-coding RNAs, and advanced engineering modifications for bone-targeted delivery in metabolic bone diseases. **Methods:** This narrative review involved a literature search conducted across PubMed, Web of Science, Scopus, and Embase from inception to December 2025, focusing on exosome biology, metabolic bone disorders, non-coding RNA biomarkers, and engineered bone-targeted delivery. Inclusion criteria encompassed peer-reviewed English original and review articles, while conference abstracts, case reports, editorials, and non-English literature were excluded. Eighty-two eligible studies underwent thematic analysis without a formal systematic search protocol or preregistration. **Results:** Exosomes orchestrate osteogenic and osteoclastogenic processes through established bone-metabolic signaling cascades. Exosomal microRNAs and circular RNAs demonstrate promising diagnostic performance in exploratory observational cohorts for early-stage screening and prognostic assessment of metabolic bone diseases; however, large-scale multi-center clinical validation remains insufficient. Engineered exosomes facilitate precise local bone intervention in preclinical animal models, overcoming significant drawbacks of conventional therapies in experimental settings. Nevertheless, clinical translation is obstructed by non-standardized production workflows and unresolved biosafety risks. **Conclusions:** Exosomes present promising dual diagnostic and therapeutic potential to enhance precision and individualized management of metabolic bone diseases. Further technical refinement in exosome preparation and modification will expedite orthopedic clinical translation and improve patient outcomes in metabolic bone disorders.

## 1. Introduction

Metabolic bone diseases disrupt bone homeostasis, encompassing osteoporosis and classic metabolic bone disorders while also addressing osteoarthritis, a degenerative joint disease characterized by significant metabolic-driven subchondral bone remodeling. Collectively, these conditions lead to substantial morbidity and disability worldwide. The International Osteoporosis Foundation reported approximately 200 million individuals living with osteoporosis globally in 2023, with one-third of women and one-fifth of men over 50 projected to experience fragility fractures in their lifetime. In 2020, osteoarthritis impacted 528 million people, with its prevalence expected to rise by 2050, positioning it among the foremost causes of global disability [1,2]. Current diagnostic methods, such as bone mineral density testing, are insensitive for early osteoporosis detection, and reliable early biomarkers for osteoarthritis remain lacking. Bisphosphonates and other first-line drugs often lead to severe long-term adverse reactions, negatively affecting patient adherence to long-term medication regimens. Osteogenic agents are further constrained by brief treatment courses and potential oncogenic risks [3]. Conventional pharmaceuticals exhibit poor targeting and bioavailability, failing to remodel the bone microenvironment and reverse pathological lesions. Existing diagnostic and therapeutic strategies display significant limitations: bone mineral density detection solely indicates irreversible bone loss in later stages, while systemic drugs are unable to concurrently regulate disordered bone metabolism, inflammation, and the immune microenvironment. Consequently, there is an urgent need for sensitive early biomarkers and targeted therapies to enhance the clinical diagnosis and treatment of patients with metabolic bone disorders. As natural nanocarriers, exosomes have shown potential in preclinical data for enabling precise diagnosis and targeted therapies, despite limited clinical validation.

Extracellular vesicles (EVs) encompass a diverse family of membrane-bound particles released by cells. Small EVs (sEVs), defined as EVs smaller than 200 nm, include exosomes, a specific subset derived from multivesicular bodies (MVBs) [4]. Many studies focused on bone research refer to isolated vesicle preparations as “exosomes” based merely on size-based separation methods, lacking definitive experimental confirmation of MVB biogenesis. For clarity, this manuscript maintains the original nomenclature from each cited publication. These vesicles carry cell-specific nucleic acids, proteins, lipids, and metabolites, accurately reflecting the status of their parent cells, thus making them ideal candidates for clinical liquid biopsy [5]. Within the bone microenvironment, exosomes facilitate communication among osteoblasts, osteoclasts, mesenchymal stem cells, and immune cells, regulating core metabolic pathways such as runt-related transcription factor 2 (Runx2)/osteoprotegerin (OPG)/receptor activator of nuclear factor-κB ligand (RANKL), Wnt, and nuclear factor-κB (NF-κB) [6]. Mechanically stimulated myoblast-derived exosomes enhance osteogenesis in bone marrow mesenchymal stem cells (BMSCs) via the miR-92a-3p/phosphatase and tensin homolog (PTEN)/protein kinase B (AKT) pathway, mitigating bone loss in glucocorticoid-induced osteoporosis [7]. Fusion exosomes derived from M2 macrophages and urine stem cells target periprosthetic osteolysis and restore bone homeostasis [8]. With low immunogenicity, high stability, and strong tissue penetration, exosomes present significant advantages for clinical diagnostic detection and targeted drug delivery in orthopedic practice [9].

Due to their unique biological properties and universal regulatory capacity in the bone microenvironment, sEVs hold dual translational potential for metabolic bone diseases. Exosomes function as both diagnostic biomarkers and therapeutic delivery systems, enabling the monitoring of disease progression and treatment responses while facilitating precise interventions in the bone microenvironment. This capability supports the shift towards personalized and integrated clinical management strategies [10,11]. Specifically, miRNAs such as miR-34a and miR-27a-3p, found in exosomes, exhibit dysregulation in early osteoporosis and may serve as potential diagnostic indicators [12]. Engineered exosomes modified with peptides can effectively deliver siRNA to osteoblasts, combining osteogenic, anti-osteoclast, and angiogenic effects [13]. Non-osteogenic sEVs are particularly suitable for clinical translation due to their excellent bone-targeting properties and diverse cell origins [14]. In summary, exosomes present innovative approaches for liquid biopsy and contribute to the development of intelligent, low-toxicity targeted therapies for metabolic bone diseases [15,16].

A comprehensive literature search was conducted across PubMed, Web of Science, Scopus, and Embase from database inception to December 2025. The search terms were organized around four core themes: exosomes/sEV, metabolic bone homeostasis and disorders, non-coding RNA biomarkers, and bone-targeted engineered delivery. Only peer-reviewed original studies and review articles published in English were included, while conference abstracts, case reports, editorials, and non-English literature were excluded. Following the screening of titles and abstracts and subsequent full-text evaluations, 82 eligible studies were included for thematic synthesis. Several articles included are Online First publications released in late 2025, which carry a 2026 formal publication year in their journal volume numbering. This review is structured as a narrative rather than a formal systematic review, aiming to provide a comprehensive, thematically organized overview of the current evidence rather than a quantitative systematic synthesis. Three key aspects of sEV/exosome-related research for metabolic bone disorders are covered: sEV-mediated biological regulation of bone homeostasis, biomarker performance of vesicle-derived non-coding RNAs, and engineering strategies for bone-targeted therapeutic delivery. Additionally, translational bottlenecks such as vesicle heterogeneity and manufacturing limitations are discussed before concluding with future perspectives.

## 2. Biological Regulatory Roles of Exosomes in Metabolic Bone Diseases

Bone homeostasis imbalance serves as the fundamental pathogenesis of metabolic bone diseases, with exosomes playing a pivotal role in regulating classic signaling pathways associated with bone metabolism. This section first elucidates the core molecular mechanisms underlying bone homeostasis and subsequently interprets the regulatory network mediated by exosomes.

### 2.1. Pathogenesis of Metabolic Bone Diseases: Bone-Homeostasis Imbalance

Bone homeostasis is achieved through a dynamic balance between osteoblast-mediated bone formation and osteoclast-dominated bone resorption. RANKL binds to its receptor, receptor activator of nuclear factor-κB (RANK), activating downstream signaling through nuclear factor of activated T-cells cytoplasmic 1 (NFATc1) to promote osteoclast differentiation and activation. OPG functions as a decoy receptor, competitively binding to RANK and preventing RANKL-induced osteoclast activation, thereby inhibiting excessive bone resorption. An elevated RANKL/OPG ratio serves as the central pathological driver of bone loss in conditions such as osteoporosis, osteoarthritis, and gouty bone erosion [17,18,19].

**Figure 1:**
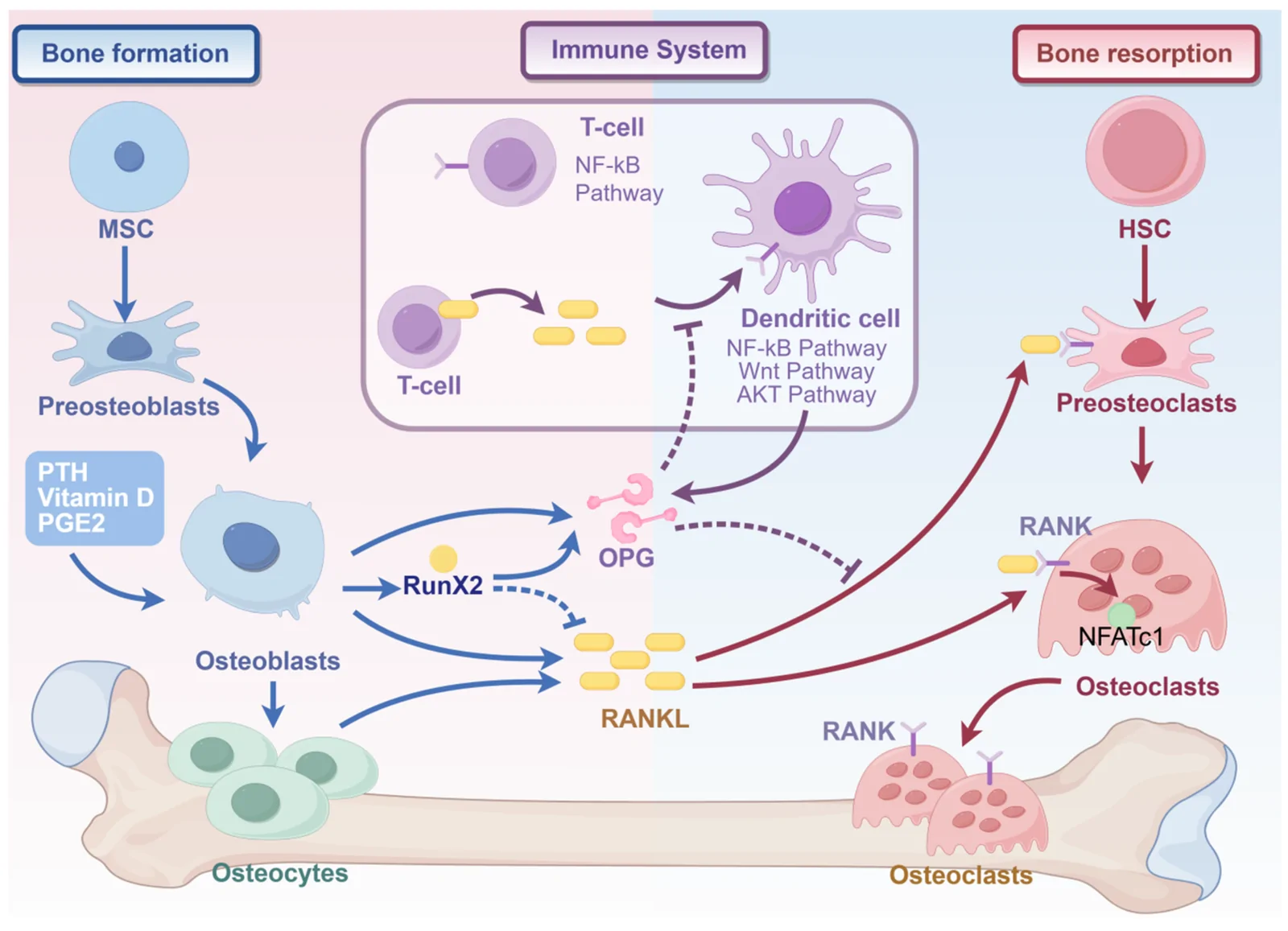
Schematic representation of the RANKL/RANK/OPG axis and immune-bone crosstalk in maintaining bone homeostasis. Osteoblasts, derived from bone marrow mesenchymal stem cells under the influence of PTH, vitamin D, PGE2, and Runx2, secrete RANKL and OPG. RANKL binds to the RANK receptor on preosteoclasts, promoting their differentiation and facilitating osteoclastic bone resorption through the NFATc1 signaling pathway. OPG, acting as a decoy receptor, competes with RANKL for binding to RANK, thus inhibiting osteoclast activation and preserving the balance between osteogenesis and resorption. In the immune system, T cells and dendritic cells modulate RANKL/OPG expression via pathways such as NF-κB and JNK, facilitating immune-bone crosstalk and contributing to the regulation of bone microenvironment homeostasis.

Runx2 is a critical transcription factor for osteogenic differentiation, regulating the expression of osteogenic markers such as type I collagen (Col1), osteocalcin (OCN), and bone morphogenetic protein 4 (BMP4), and closely interacting with the OPG-RANKL axis [20]. In ovariectomized osteoporotic rats, combined treatment with metformin and total flavonoids from Rhizoma Drynariae enhances bone mineral density and trabecular microarchitecture by upregulating Runx2 and lowering the RANKL/OPG ratio [21]. Similarly, Resolvin D1 facilitates alveolar bone osseointegration and regeneration through Runx2 upregulation and suppression of RANKL/OPG signaling [22]. Various bioactive substances—dietary components, vitamins, and herbal polysaccharides—have been shown to restore the bone metabolic balance by targeting this axis [23,24,25,26]. Furthermore, the OPG-RANKL-RANK axis also participates in inflammatory responses related to obesity, type 2 diabetes, and non-alcoholic fatty liver disease, indicating its systemic regulatory role in metabolism-associated bone disorders (Figure 1) [27,28].

### 2.2. Exosome-Mediated Intercellular Communication in Bone Microenvironment

Exosomes play a critical role in delivering functional non-coding RNAs and proteins, facilitating cell communication that regulates bone formation, resorption, immune crosstalk, and angiogenesis, thereby maintaining bone homeostasis [15,29]. Exosomes secreted by BMSCs, osteoblasts, and macrophages are essential for stabilizing bone metabolism [30]. For example, the polarization state of macrophages influences exosome function: M1 macrophage-derived exosomes promote bone resorption via the NF-κB pathway, whereas M2 macrophage exosomes support bone repair [31].

Exosomes also modulate osteogenic progression through various molecular mechanisms. Exosomal long non-coding RNA (lncRNA) NEAT1, derived from prostate cancer cells, upregulates Runx2 to facilitate the osteogenic differentiation of human BMSCs. Additionally, myoblast-derived exosomes enhance BMSC proliferation and osteogenesis through the miR-92a-3p/PTEN/AKT and Prrx2/MIR22HG/miR-128/YAP pathways, alleviating bone loss in models of glucocorticoid- and ovariectomy-induced osteoporosis [7,32].

Under pathological conditions, dysregulated exosomal signaling can disrupt bone homeostasis and exacerbate bone damage. For instance, senescent BMSC-derived exosomes promote osteoclast formation, leading to increased bone resorption and implant failure [33]. In gout, neutrophil-derived exosomal miR-1246 alters RANKL and OPG expression and compromises osteoblast activity, resulting in bone erosion [17].

**Figure 2:**
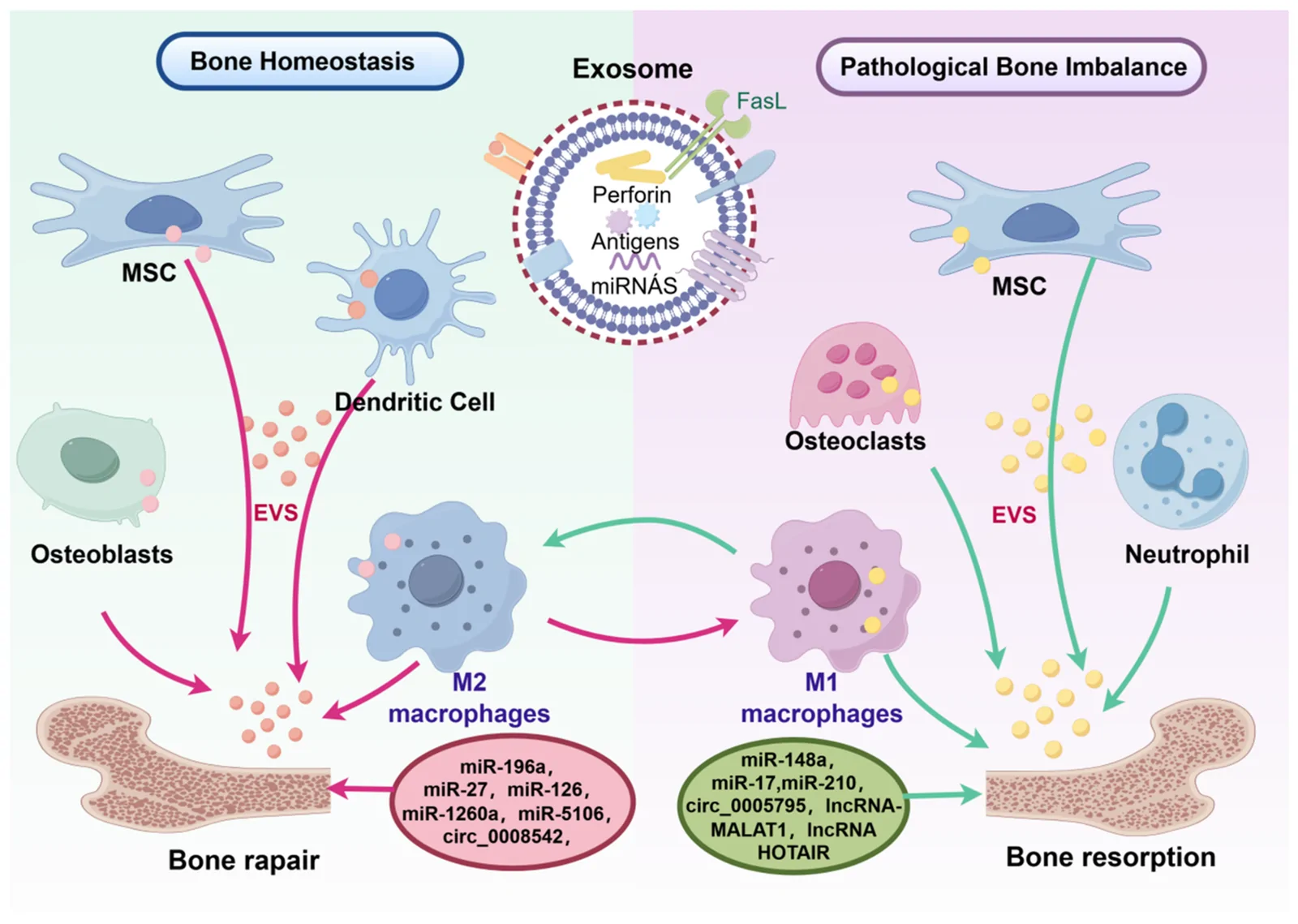
Schematic representation of exosome-mediated cell-to-cell communication in bone homeostasis and pathological bone imbalance. (**Left**) Bone homeostasis and repair. Under physiological conditions, mesenchymal stem cells, osteoblasts, dendritic cells, and M2 macrophages release homeostatic exosomes that carry bone-protective miRNAs. These exosomes mediate osteoimmunological interactions, promoting osteoblast-driven bone formation and repair. (**Right**) Pathological bone imbalance. In pathological conditions, MSCs, neutrophils, osteoclasts, and M1 macrophages secrete pathogenic exosomes containing bone-resorptive miRNAs, triggering osteoclast-mediated bone resorption and exacerbating inflammatory bone loss.

Exosomes function as key mediators of intercellular signals and regulators of bone metabolic disorders (Figure 2). Their heterogeneity enables precise disease subtyping and individualized therapeutic approaches. Future studies are required to elucidate the functions of different exosome subpopulations and investigate their interactions with gut microbiota and the nervous system. The functional outputs of exosomes are significantly influenced by their heterogeneity, which arises from donor phenotype, disease context, and vesicle subpopulations (Section 5.2) [4].

### 2.3. Inflammatory-Microenvironment-Driven Functional Reprogramming of Exosomes

#### 2.3.1. Inflammation Triggers Pathological Reprogramming of Exosomes

Chronic inflammation serves as a core pathological driver of metabolic bone disorders, with persistent inflammatory stimuli capable of remodeling the cargo composition of exosomes and shifting their biological functions toward bone destruction [34]. Specifically, pro-inflammatory cytokines, such as tumor necrosis factor-α (TNF-α) and interleukin-6 (IL-6), alter the characteristics of exosome-releasing cells and modify exosomal components, transforming exosomes from bone-protective to bone-destructive phenotypes [35]. Within the inflamed bone microenvironment, these altered exosomes activate macrophage-mediated NF-κB/RANKL signaling, facilitating osteoclast differentiation while simultaneously inhibiting Wnt/Runx2-dependent osteogenic activity, ultimately leading to progressive bone loss [36].

Supporting this pathogenic regulatory pattern, monosodium urate (MSU) crystals in gout trigger activated neutrophils to release exosomes enriched with miR-1246. These exosomes suppress osteoblastic alkaline phosphatase (ALP) and OPG expression while elevating RANKL levels, demonstrating that inflammatory stimulation modifies exosomal cargo to exacerbate bone erosion [17]. In addition to inflammatory stimuli, cellular stress signals can also be transmitted via exosomes, disrupting balanced bone remodeling.

#### 2.3.2. Therapeutic Exosomes Reverse Inflammatory Bone Imbalance

In contrast to the pathogenic exosomes induced by inflammatory stress, therapeutic exosomes or exosome-modulating agents can restore disrupted bone remodeling homeostasis, reflecting the dual regulatory characteristics of exosomes within inflammatory microenvironments.

A series of preclinical in vivo studies have demonstrated protective effects in models of metabolic bone diseases. For instance, macrophage-derived sEVs polarized toward the M2 phenotype can dampen local inflammatory cascades: these vesicles suppress NF-κB-driven pro-inflammatory signaling, promote osteoblast viability, and mitigate pathological bone resorption in ovariectomized osteoporotic mice [37]. Additionally, sEVs secreted by adipose-derived stem cells and enriched with miR-146a can skew both synovial and bone-resident macrophages toward an anti-inflammatory phenotype, limiting inflammatory bone erosion in collagen-induced arthritis models [38]. Furthermore, bone marrow-derived sEVs also exhibit potent anti-inflammatory and bone-protective bioactivity. Delivered through sEV-encapsulated miR-29b-3p, these vesicles suppress NF-κB-driven inflammatory cascades and reduce pathological bone loss in ovariectomized osteoporotic mice [39].

**Figure 3:**
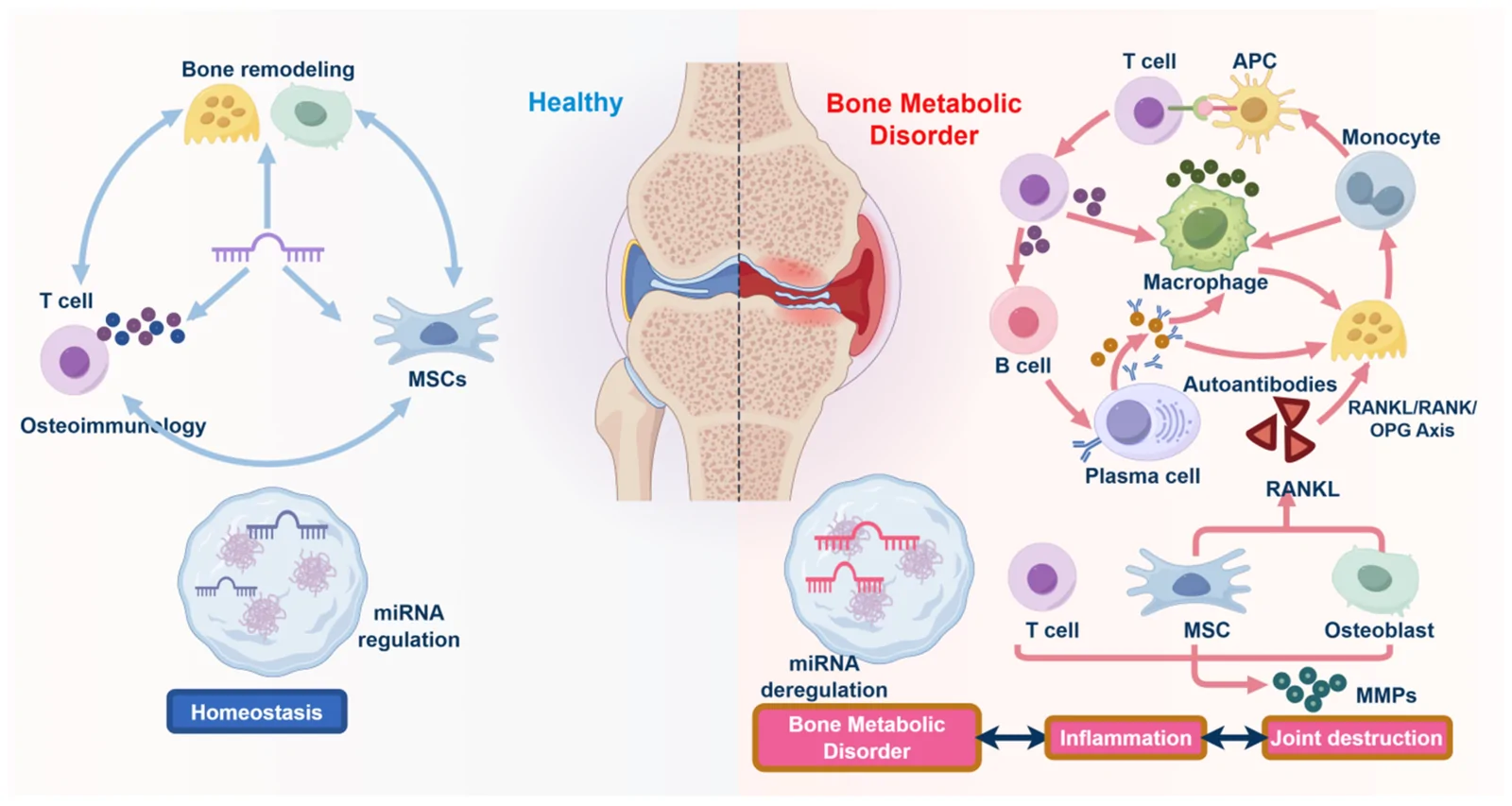
Schematic representation of exosome functional reprogramming in inflammatory bone metabolic disorders. In healthy bone homeostasis, exosomes regulate a balanced network of immune cells, osteoblasts, and osteoclasts, facilitating the synchronized coupling of bone formation and resorption. In a chronic inflammatory bone microenvironment, pro-inflammatory cytokines induce cargo reprogramming within exosomes, shifting their function from bone-protective to bone-destructive phenotypes. This pathogenic cascade involves dysregulated immune cells, disruption of the RANKL/RANK/OPG signaling pathway, excessive osteoclast activation, and suppressed osteoblast function, collectively creating a self-amplifying cycle that drives persistent bone destruction and metabolic imbalance.

In conclusion, inflammation can utilize sEV-mediated signaling to exacerbate bone lesions (Figure 3). Conversely, the functional plasticity of sEVs creates new avenues for targeted therapy using engineered sEV platforms. The bidirectional crosstalk between sEVs and inflammation represents a promising research direction for the immunotherapy of bone diseases. Importantly, as circulating intercellular messengers, sEVs facilitate communication between different organs and contribute to the formation of an “intestine-metabolism-bone” regulatory axis, which offers a novel perspective for exploring systemic metabolic bone disorders.

## 3. Exosomes as Biomarkers for Metabolic Bone Diseases

### 3.1. Exosomal Non-Coding RNAs as Candidate Diagnostic Biomarkers

Chronic inflammation and bone metabolic disorders significantly modify the cargo profiles of exosomes. Exosomal miRNAs and circRNAs, due to their high stability and specific expression patterns, play critical regulatory roles in bone metabolism and are thus considered promising diagnostic biomarkers for early clinical screening of metabolic bone diseases [40]. These non-coding RNAs specifically regulate oxidative stress and the balance between osteoblasts and osteoclasts, contributing to the progression of osteoporosis. For instance, circ-0091579 is upregulated, while circ-HIPK3 is downregulated in postmenopausal osteoporosis patients, with their expression levels closely correlating with inflammatory and bone metabolic indicators, effectively distinguishing healthy individuals from those with osteopenia and osteoporosis [41]. Furthermore, various exosomal miRNAs are also involved in the regulation of bone homeostasis. BMSC-derived miR-21-5p targets SKP2 to stabilize FoxO1, thereby promoting osteogenesis and mitigating age-related bone loss [42]. Similarly, plasma exosomal miR-142-5p, which is highly expressed in younger populations, can enhance osteogenesis and inhibit osteoclastogenesis, indicating its potential as an early diagnostic marker [43]. However, many of these non-coding RNA candidates have been identified through preclinical mechanistic studies, with relatively few formally assessed as diagnostic biomarkers in human cohorts. Clinical metadata, including patient cohort characteristics and vesicle isolation protocols, is often incompletely detailed in original publications (see footnote under Table 1).

In addition to primary osteoporosis, abnormal expression of exosomal miRNAs also contributes to various forms of secondary osteoporosis. Lead exposure induces the upregulation of exosomal miR-30a-3p, triggering osteoblast pyroptosis via the NOD-like receptor pyrin domain-containing protein 3 (NLRP)/caspase-1 pathway [44]. In ovariectomized animal models, decreased expression of miR-29a-3p and miR-29c-3p increases tet methylcytosine dioxygenase 3 (TET3), causes hypermethylation of the Sox9 promoter, and inhibits the phosphatidylinositol 3-kinase (PI3K)/AKT pathway, ultimately suppressing osteogenic differentiation [45]. Additionally, dysregulated miR-151-3p and miR-23b-3p in BMSC exosomes further disrupt bone metabolic balance [46]. Finally, BMSCs stimulated by FeO nanoparticles secrete exosomes enriched with miR-1260a, which can repress histone deacetylase 7 (HDAC7) and COL4A2, facilitating osteogenesis and angiogenesis (Table 1) [47].

Individual exosomal non-coding RNAs, such as circ-0091579 and miR-142-5p, exhibit diagnostic potential; however, their performance as standalone biomarkers is limited in the context of complex metabolic bone diseases. Factors restricting their efficacy include biological heterogeneity, fluctuating expression levels, and cross-expression in other disorders. The pathogenesis of osteoporosis involves the imbalance between bone formation and resorption, inflammatory responses, and intricate intercellular communication, making it inadequate for a single non-coding RNA to fully encapsulate such multidimensional pathological information.

Consequently, constructing combined biomarker panels consisting of multiple exosomal miRNAs and circRNAs emerges as an effective strategy to significantly enhance the accuracy of early diagnosis and disease classification, facilitating precise clinical staging of metabolic bone diseases. By integrating molecular data that reflect distinct pathological pathways, multi-biomarker combinations can create comprehensive and specific “molecular fingerprints.” Several studies have demonstrated that combined circulating miRNA panels can improve diagnostic accuracy for osteoporosis [48,49]. Similarly, rationally combining non-coding RNAs associated with osteogenic activity, osteoclastic activity, and inflammatory status enables a more accurate evaluation of the severity and pattern of bone metabolic disorders. Furthermore, these combined panels allow differentiation among disease stages and subtypes, with distinct expression profiles of circRNAs and miRNAs effectively distinguishing healthy individuals from those with osteopenia and osteoporosis [50], thereby establishing a strong foundation for refined disease classification and personalized therapy. Future research should prioritize screening and validating optimal combined non-coding RNA biomarker panels through large-scale clinical cohorts and advanced bioinformatics analysis.

### 3.2. Stratified Evidence Evaluation of Exosome-Based Biomarkers

Current evidence can be categorized into three major groups: preclinical animal studies, human observational biomarker analyses, and interventional clinical trials [51]. To date, no completed interventional clinical trials have assessed exosome-based therapeutics for metabolic bone disorders [51,52]. Most mechanistic insights stem from rodent osteoporosis models, including ovariectomy-induced osteoporosis, glucocorticoid-induced osteoporosis, and gout-associated bone erosion, typically involving 6–15 animals per experimental group [53]. Human biomarker evidence is largely limited to small- to moderate-sized observational case–control and cross-sectional cohorts, primarily including postmenopausal women and comparing healthy controls with individuals diagnosed with osteopenia or osteoporosis [54,55]. The total sample size of these cohorts ranges from approximately 110 to 140 participants. Several observational studies report ROC-derived diagnostic performance; combined exosomal miRNA panels achieve AUC values between 0.80 and 0.92 for differentiating postmenopausal osteoporosis patients from healthy controls, while individual exosomal miRNAs reach AUC values between 0.69 and 0.82 in single-center cohorts [54,55]. Nonetheless, many observational studies only document differential molecular expression profiles without calculating formal diagnostic parameters such as AUC, sensitivity, or specificity [55,56]. Additionally, nearly all existing diagnostic data derive from single-center populations, necessitating independent multicenter validations before real-world clinical translation (Table 2) [55,57].

## 4. Engineering Strategies for Bone-Targeted Exosomes

**Figure 4:**
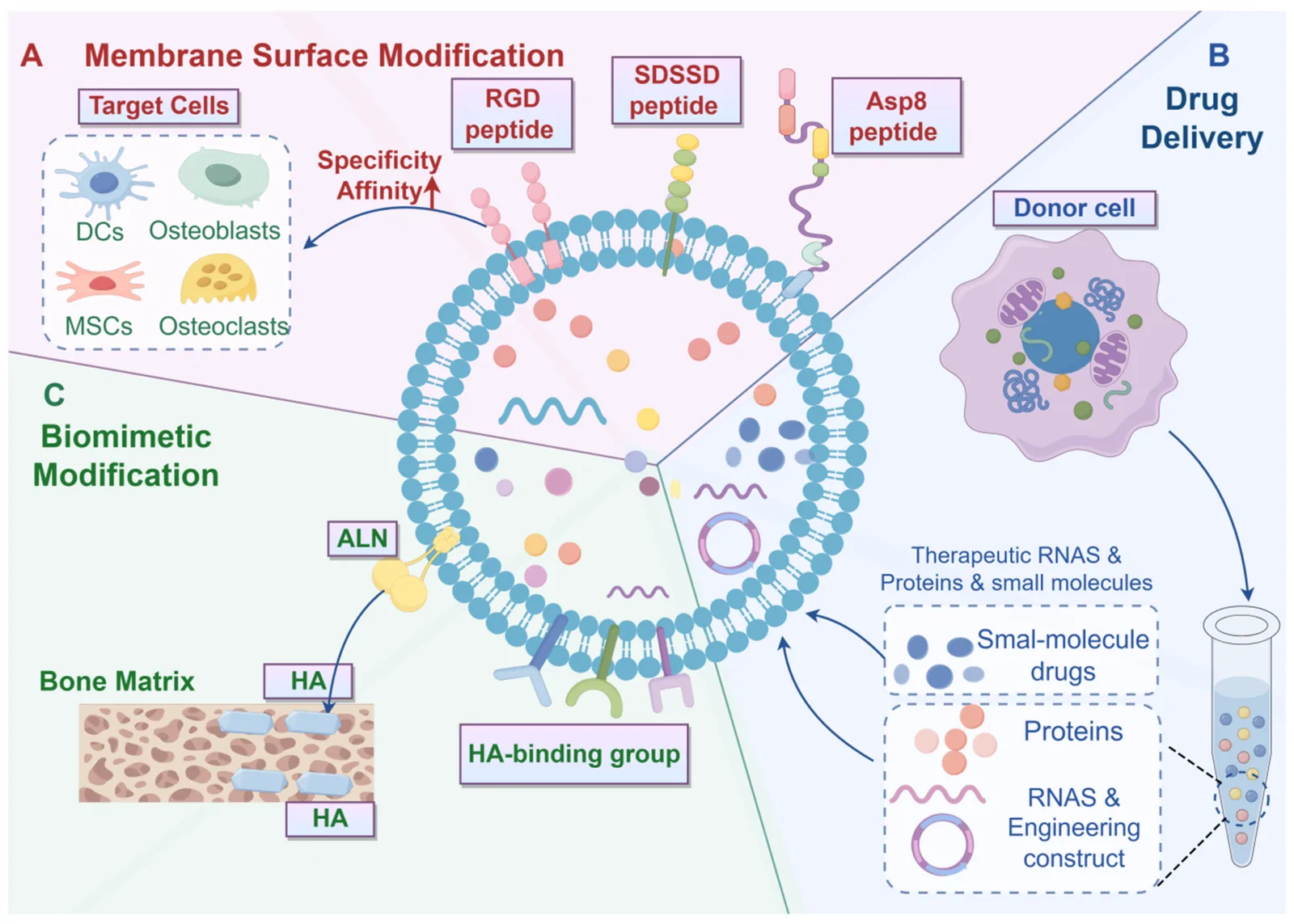
Schematic illustration of engineering strategies for bone-targeted exosome modification and drug delivery. (**A**) Membrane surface modification: Bone-targeting ligands, such as RGD peptide, SDSSD peptide, and Asp_8_ peptide, are conjugated to the exosomal membrane to enhance specificity and affinity for bone-related cells. (**B**) Drug delivery system: Exosomes function as intelligent carriers, efficiently loading and delivering therapeutic agents including small-molecule drugs, proteins, and nucleic acids. (**C**) Biomimetic modification: Bisphosphonates (e.g., alendronate, ALN) and hydroxyapatite (HA)-binding groups are incorporated to improve bone homing, enabling targeted accumulation in the bone matrix and defect sites to promote osteogenic differentiation and bone regeneration.

As exosomal non-coding RNAs have emerged as promising diagnostic biomarkers, engineered modifications have become essential for enhancing exosomes’ targeting performance and therapeutic functions in metabolic bone diseases. This section summarizes the mainstream strategies for bone-targeted engineering of exosomes (Figure 4).

### 4.1. Membrane Surface Modification: RGD Peptides and Bone-Targeting Aptamers

The bone matrix primarily consists of hydroxyapatite, which provides specific anchoring sites for targeted delivery vectors. Compared to large-molecule antibody modifications, short bone-targeting peptides offer distinct advantages, including low immunogenicity, ease of synthesis, and excellent biocompatibility, making them ideal candidates for exosome functional engineering. Representative targeting motifs such as Asp_8_, SDSSD, and Arg-Gly-Asp (RGD) specifically recognize bone mineral components or surface receptors on bone-resident cells [16]. Among these ligands, RGD peptides and bone-targeting aptamers demonstrate the highest affinity and specificity for bone tissue. RGD sequences selectively bind to integrins that are highly expressed in the bone microenvironment, substantially enhancing exosome adhesion to osteoblasts and the bone matrix.

RGD-functionalized exosomes activate the focal adhesion kinase (FAK)-Src-AKT osteogenic signaling cascade. When combined with inherent exosomal bioactive cargos, this pathway activation synergistically upregulates key osteogenic transcription factors (Runx2, Osterix), suppresses RANKL expression, and increases OPG levels, thereby restoring the osteogenic-osteoclastic balance and facilitating bone reconstruction for clinical treatment of bone repair [13]. Similarly, bone-targeting aptamers enable precise exosome anchoring to bone lesion sites due to their high specificity, low molecular weight, and modifiable properties [60,61]. Overall, membrane surface modification strategies effectively enhance exosome accumulation and retention in bone tissue, compensating for the inadequate targeting capability of natural exosomes. This results in precise, localized regulation of the pathological bone microenvironment, which helps to mitigate systemic side effects of clinical medications. Consequently, functionally engineered exosomes exhibit superior therapeutic performance and translational potential for metabolic bone diseases compared to their unmodified counterparts.

### 4.2. Cargo Loading Engineering for Functional Exosome Reinforcement

Natural exosomes possess inherent therapeutic potential but are limited by insufficient cargo abundance, non-specific payload distribution, and restricted regulatory efficacy, hindering their standalone application in severe bone metabolic disorders. Cargo loading engineering allows for precise packaging of therapeutic nucleic acids, proteins, and small-molecule drugs into exosomes, thereby enhancing their biofunctional performance and imparting programmable therapeutic characteristics [62,63]. Current loading strategies are primarily divided into endogenous pre-loading and exogenous post-loading approaches.

Endogenous pre-loading involves manipulating parental cells to overexpress target functional molecules, which are subsequently encapsulated into secreted exosomes during biogenesis. This method ensures high biocompatibility and stable cargo embedding, avoiding structural damage to exosome membranes. Multiple studies have utilized this approach to enrich BMSC-derived exosomes with osteogenic miRNAs and anti-inflammatory proteins, which persistently activate Wnt/β-catenin signaling, inhibit NF-κB-mediated inflammation, and effectively mitigate bone loss in osteoporotic models [64,65]. However, endogenous loading is constrained by low cargo yield and uncontrollable packaging efficiency, making standardized mass production challenging.

In contrast, exogenous post-loading directly incorporates therapeutic agents into isolated exosomes using physical or chemical methods, including electroporation, ultrasonic treatment, freeze–thaw cycling, and chemical transfection. This strategy offers high loading efficiency, flexible cargo selection, and controllable dosage, making it more suitable for standardized therapeutic preparations. Electroporation is the most commonly used technique, creating transient membrane pores that facilitate the internalization of siRNA, miRNA, and drugs without compromising exosome integrity. Exosomes loaded with osteogenic siRNA or anti-osteoclastic miRNAs achieve targeted modulation of the Runx2/OPG-RANKL axis, promoting bone formation while simultaneously suppressing excessive bone resorption [13]. Despite its advantages, exogenous loading may induce minor membrane damage and cargo leakage, necessitating optimized parameter calibration to ensure exosome stability and biological activity. Additionally, co-loading multiple therapeutic molecules is an emerging trend, enabling exosomes to exert synergistic effects on osteogenesis, anti-inflammation, and anti-osteoclastic activity.

### 4.3. Biomimetic Strategies for Enhanced Exosome Bone Homing

The aforementioned membrane surface modification and cargo loading technologies enhance the targeting ability and therapeutic efficacy of exosomes. However, conventional chemical modifications may invoke potential immune responses. As a novel technical approach, biomimetic modification addresses this limitation and further improves the bone-homing efficiency of exosomes. Recent advancements in biomimetic modification strategies have significantly enhanced the bone-homing efficiency and therapeutic efficacy of engineered exosomes by mimicking natural biological processes and the physicochemical properties of bone tissue. This innovation establishes a new paradigm for bone-targeted modification. For instance, bisphosphonates (e.g., ALN) are employed to modify the exosome surface, leveraging their high affinity for hydroxyapatite to achieve bone targeting. Exosomes derived from platelet lysates, when modified with ALN (designated as PL-exo-ALN), exhibit enhanced hydroxyapatite binding in vitro and effectively accumulate in the bone tissue of glucocorticoid-induced osteoporosis mice post intravenous injection, simultaneously promoting osteogenesis and angiogenesis [66]. Another study introduced hydroxyapatite-binding groups onto the membrane of biomimetic exosomes (EMs) using bioorthogonal chemistry, allowing these exosomes to specifically accumulate at bone defect sites following systemic administration. The EM/SAG complex, loaded with a smoothened agonist (SAG), significantly promotes osteogenic differentiation and accelerates bone regeneration in vivo [67].

Moreover, natural exosomes derived from mineralized osteoblasts (MOBs) possess inherent bone-targeting potential. When combined with 3D-printed hydroxyapatite scaffolds, these exosomes facilitate osteogenic mineralization of BMSCs without the need for additional inducing factors [68]. sEVs released by neurons after trauma naturally target bone tissue due to the upregulation of fibronectin on their surface, suggesting that biomimetic modification of this protein could further enhance the bone-homing capacity of exosomes [69]. These advances indicate that biomimetic modification strategies not only circumvent potential immune reactions associated with traditional chemical modifications but also maximize the unique physicochemical microenvironment of bone tissue, providing a novel approach for constructing efficient and safe bone-targeted exosome delivery systems for future clinical translation [16,31].

In summary, the three bone-targeted engineering strategies discussed complement one another with distinct advantages. Surface peptide and aptamer modifications primarily enhance the enrichment of exosomes in bone tissue, while cargo loading engineering strengthens therapeutic efficacy by enriching functional nucleic acids and drugs. Biomimetic modification mitigates potential immune side effects associated with chemical conjugation, demonstrating a favorable biosafety profile observed in preclinical studies for long-term in vivo administration. The combined use of surface targeting and multi-cargo co-loading may represent the mainstream optimization direction for bone-targeted exosomes in the future. However, these strategies may not be equally suitable for all applications. A systematic comparison of their respective performance and translational characteristics is detailed below (Table 3).

### 4.4. Comparative Evaluation of Bone-Targeted Engineering Strategies

Bone-targeted membrane modification, cargo-loading workflows, and biomimetic approaches each enhance exosome performance for metabolic bone disease research; however, each technique also possesses specific limitations.

Peptide- and aptamer-based ligands effectively bind to bone minerals and are inexpensive to synthesize, carrying minimal immunogenic risk [70]. Their primary drawback occurs in circulation, where short peptides and aptamers are susceptible to degradation by circulating enzymes. Additionally, RGD-based constructs may bind off-target, as integrins are expressed beyond bone tissue [70,71]. Furthermore, surface conjugation does not introduce therapeutic cargo; exosomes require a separate loading step to achieve therapeutic activity.

For cargo loading, endogenous pre-loading involves integrating payloads into exosomes during parental cell biogenesis. While this method ensures stable integration and biocompatibility, it often yields low and variable packaging levels, complicating standardized large-scale production [72]. Conversely, exogenous post-loading techniques, such as electroporation, sonication, and freeze–thaw cycles, accommodate a wider range of payloads and achieve higher loading capacities, albeit at the expense of membrane integrity. Electroporation, in particular, can result in the aggregation of nucleic acids, diminishing the fraction of functionally encapsulated cargo [72,73]. Both loading methods lack bone-homing moieties, necessitating additional surface modification for effective bone targeting [71].

Bisphosphonate-based biomimetic modification leverages the strong affinity of bisphosphonates for hydroxyapatite, allowing selective bone accumulation with reduced off-target exposure [74]. Given that ALN is already utilized clinically, the regulatory profile of its conjugates is relatively well understood. However, scalability depends on the conjugation chemistry: reproducible chemical labeling can be scaled effectively, while exosomes harvested from mineral-producing cells present challenges for consistent large-scale production. Additionally, residual unconjugated bisphosphonates may exert independent pharmacological effects [71,74].

In practical experimental or translational settings, researchers must consider intended use, cargo physicochemical properties, and manufacturing constraints when selecting modification workflows. Combinations that integrate bone-targeting surface decoration with optimized cargo loading present a viable approach to balancing potency, safety, and manufacturability.

## 5. Clinical Translation Progress and Limitations of Exosome-Based Diagnosis and Therapy

A variety of bone-targeted modification and cargo loading techniques have significantly enhanced the performance of exosomes. Based on these technological advances, exosomes can be utilized for clinical diagnosis and intervention in metabolic bone diseases. This summary evaluates the current application status, advantages, and primary limitations of exosome-based diagnostic and therapeutic approaches.

### 5.1. Preclinical and Clinical Advances of Engineered Exosome Therapeutics

Most efficacy data for engineered exosome therapeutics in metabolic bone diseases have been derived from preclinical animal models. To date, no completed human interventional clinical trials have been published for engineered exosome therapies targeting osteoporosis [51]. Engineered exosomes have demonstrated notable preclinical efficacy across multiple metabolic bone disease models, including osteoporosis, osteoarthritis, and inflammatory bone erosion, thereby establishing a strong foundation for forthcoming clinical trials [75]. Bone-targeted engineered exosomes selectively accumulate in damaged bone tissue, regulate key bone metabolic signaling pathways, and mitigate pathological bone loss with minimal systemic toxicity observed in preclinical models, showing advantages over conventional systemic drugs in these experimental settings [76]. Cargo-optimized exosomes facilitate synergistic osteogenic, anti-inflammatory, and anti-osteoclastic effects, enabling multi-target collaborative intervention within the pathological bone microenvironment [77]. Additionally, fusion exosomes enhance therapeutic stability and functional diversity, presenting innovative strategies for treating challenging bone metabolic disorders [75].

Despite significant preclinical progress, several bottlenecks impede the clinical translation of exosome therapeutics. First, large-scale standardized production remains a challenge, as traditional cell culture-based exosome extraction often results in low yield, high batch-to-batch variability, and insufficient purity, which do not meet clinical dosage and quality consistency requirements. Second, the absence of unified quality control systems limits progress. No consensus exists on exosome identification criteria, activity detection indicators, and cargo quantification standards, hindering industrial preparation efforts [78]. Third, in vivo delivery efficiency requires further enhancement. Although engineered modifications improve bone targeting, systemic circulation clearance and non-specific tissue uptake still restrict local therapeutic concentrations [16]. Finally, long-term biosafety evaluations are inadequate. Heterogeneity stemming from donor sources, isolation workflows, and cargo variations complicates consistent biosafety assessments. The potential immunogenicity, off-target effects, and pathological risks associated with repeated exosome administration necessitate thorough long-term verification [75]. Additionally, the separation and identification of exosome subpopulations are crucial for industrialization, facilitating precise medication and uniform therapeutic outcomes.

### 5.2. Sources and Consequences of Exosome Heterogeneity; Mitigation and Standardization Strategies

Exosome heterogeneity poses a complex challenge that influences biological activity, targeting performance, and biosafety [75]. Variability arises from multiple interconnected sources. The originating donor cell type determines surface markers, lipid profiles, and intrinsic functional potency; for instance, exosomes derived from M2 macrophages exhibit anti-inflammatory and pro-osteogenic effects, while those from M1 macrophages promote bone resorption [79]. Additionally, the disease or inflammatory status of the donor material can remodel exosome cargo, potentially shifting vesicle function toward bone-destructive phenotypes and introducing unforeseen risks for therapeutic use [80]. Laboratory isolation workflows contribute further to technical heterogeneity: ultracentrifugation, size-exclusion chromatography, and precipitation-based methods yield preparations of varying purity, often containing co-isolated protein aggregates and mixed vesicle populations, which partly explain the poor reproducibility observed between laboratories [81]. Even when starting from identical donor material, bulk preparations combine functionally divergent particle subpopulations. Some subsets may possess bone-adhesive surface moieties, while other fractions lack targeting capacity; unfractionated samples may also contain vesicles loaded with pro-inflammatory cargo, presenting latent safety risks [6]. Cargo composition introduces another layer of variability, affecting osteogenic, anti-osteoclastic, and immunomodulatory outputs in both native and engineered exosome products [82].

Several practical measures can help mitigate these heterogeneities to support reproducible preclinical research and future clinical-grade manufacturing. Preference should be given to well-characterized donor cell lines cultured under tightly controlled conditions. Isolation and characterization workflows must be harmonized according to MISEV2018 reporting standards, including particle quantification, size distribution, marker profiling, and contaminant assessment [4]. Immunoaffinity enrichment can effectively isolate functionally desirable subpopulations while depleting harmful vesicle fractions. Batch-wise quality control should encompass quantitative cargo profiling, cell-based functional potency assays, and immunogenicity risk screening. For engineered constructs, consistent cargo-loading efficiency must be validated for each batch.

## 6. Discussion

As key mediators of intercellular communication, exosomes have been shown to regulate multiple core signaling pathways involved in bone metabolism. Their unique advantages allow them to function as reliable liquid biopsy biomarkers and intelligent targeted carriers, opening new avenues for the diagnosis and treatment of metabolic bone diseases in clinical orthopedics. Despite the encouraging preclinical findings, several translational and fundamental challenges continue to hinder the large-scale clinical application of exosome-based diagnostic and therapeutic strategies.

Technical standardization represents the most significant barrier to industrial transformation and large-scale clinical application. Existing production protocols lack unified criteria, necessitating the urgent integration of microfluidics and synthetic biology to establish stable, large-scale exosome manufacturing systems along with universal quality control standards for clinical products. Innovations in dual-target modification and multi-cargo co-loading technologies can further enhance the in vivo targeting capabilities of exosomes, thereby facilitating integrated diagnostic and therapeutic applications. Additionally, in-depth exploration of the crosstalk between exosomal signaling, gut microbiota, and the neuro-immune axis can contribute to a comprehensive understanding of the systemic pathogenesis of metabolic bone disorders. With the assistance of artificial intelligence and molecular docking simulations, the research cycle for engineered exosomes can be significantly accelerated, and rigorous multicenter trials are essential to provide credible evidence for the long-term safety and efficacy of exosome products.

Moreover, exosomes exhibit stage-specific dynamic effects during disease progression, indicating that personalized therapeutic strategies rather than universal treatment protocols are necessary. The potential synergies between exosome delivery systems and traditional clinical drugs remain an underexplored area. Moving forward, the research paradigm must shift to regard exosomes as dynamic systemic signal regulators rather than merely passive delivery vehicles. Continued efforts in this direction will help to fully realize their potential value in the comprehensive clinical management of metabolic bone diseases.

## 7. Conclusions

Metabolic bone diseases are characterized by disrupted bone homeostasis and chronic inflammation, creating an urgent need for reliable early diagnostics and low-toxicity targeted therapies. As natural intercellular nanocarriers, exosomes serve as powerful biomarkers for early detection and prognostic monitoring. Leveraging diverse modification technologies, engineered exosomes overcome the inherent limitations of their native counterparts, effectively regulating bone metabolism and local inflammatory status.

This narrative review synthesizes exosome-mediated regulatory mechanisms, the diagnostic value of non-coding RNA cargo, and advanced bone-targeted engineering approaches. Accumulated preclinical data highlight the considerable promise of exosome-based strategies with dual diagnostic and therapeutic potential for precision management of metabolic bone diseases. Further studies focusing on exosome heterogeneity and combined therapeutic approaches will also facilitate their clinical integration and benefit a broader patient population.

## Figures and Tables

**Table 1:** Exosomal non-coding RNAs as potential diagnostic biomarkers for metabolic bone diseases.

Molecule	Sample/Model Source	Expression Change	Mechanism & Functional Role	Reference
circ-HIPK3	PBMC	Down- regulated	Correlates with bone metabolic indicators; available as an auxiliary biomarker for postmenopausal osteoporosis	[41]
circ-0091579	PBMC	Up- regulated	Associated with bone metabolic and inflammatory markers; distinguishes postmenopausal osteoporosis patients from osteopenic and healthy subjects	[41]
miR-21-5p	MSC-derived exosomes	Highly enriched	Targets SKP2/FoxO1 axis to promote osteogenesis and alleviate age-related bone loss	[42]
miR-142-5p	Plasma exosomes	Higher in young adults	Dual regulation of enhanced osteogenesis and suppressed osteoclastogenesis, promising early diagnostic marker	[43]
miR-30a-3p	Osteoclast-derived exosomes	Up-regulated	Triggers NLRP3/caspase-1-dependent osteoblast pyroptosis to induce lead-related bone loss	[44]
miR-29a-3p/29c-3p	Serum exosomes	Down-regulated	Elevates TET3 to hypermethylate Sox9 and restrain PI3K/AKT, inhibiting osteogenic differentiation	[45]
miR-151-3p	MSC-derived exosomes	Dysregulated	Participates in multi-pathway modulation of physiological bone metabolism balance	[46]
miR-1260a	FeO + magnetic field-treated MSC exosomes	Up-regulated	Inhibits HDAC7 and COL4A2 to synergistically improve osteogenesis and angiogenesis	[47]

Note: Inclusion criteria for Table 1. Eligible entries include exosome-derived non-coding RNAs with documented expression changes associated with metabolic bone-related phenotypes. This table provides a representative summary rather than a comprehensive systematic-review-style inventory of all published candidates. Most vesicle preparations described as “exosomes” in the original studies were isolated using size-based methods without definitive evidence of multivesicular body origin and therefore correspond to sEVs according to MISEV2018 [4].

**Table 2:** Stratified summary of available evidence for exosome-related biomarkers and therapeutics in metabolic bone diseases.

Evidence Level	Representative Example	Disease Model/Population	Sample Size	Reported Outcome Parameters	Reference
Pre-clinical (animal)	BMSC-derived exosomes improve bone mass in ovariectomy-induced osteoporosis	Rat OVX-induced osteoporosis	Rat, *n* = 8–10 per group	Elevated BMD, BV/TV, Tb.N; reduced Tb.Sp; restored Runx2/OPG-RANKL balance	[53]
Pre-clinical (animal)	hiPSC-MSC-derived exosomes combined with β-TCP scaffold repair osteoporotic calvarial defects	OVX-rat critical-size calvarial bone defect	Rat, *n* = 6–8 per group	Enhanced bone regeneration and angiogenesis; dose-dependent osteogenic effect	[58]
Clinical-observational (biomarker)	Plasma exosomal 3-miRNA signature (let-7d-3p, miR-24-3p, miR-550a-3-5p)	Postmenopausal osteoporosis versus healthy controls	Total *n* = 126	Individual miRNA AUC: 0.84, 0.80, 0.82; combined panel improved diagnostic performance	[54]
Clinical-observational (biomarker)	Serum exosomal miR-9-5p and miR-320d	Postmenopausal osteoporosis and healthy subjects	Total *n* = 118	miR-9-5p AUC = 0.695 (sensitivity 73.08%, specificity 61.90%); miR-320d AUC = 0.695 (sensitivity 76.92%, specificity 57.14%)	[55]
Clinical-observational (biomarker)	Circulating 19-miRNA OsteomiR^®^ panel (four miRNA clusters)	Postmenopausal osteoporosis vs. non-osteoporotic controls	Total *n* = 100	19-miRNA panel AUC = 0.830 (WHO osteoporosis diagnostic criteria); AUC = 0.834 (major osteoporotic fracture history)	[59]

This table summarizes representative animal experiments and observational biomarker studies in exosome-related osteoporosis research. Preclinical studies primarily rely on ovariectomized rodent models. Available clinical diagnostic data are derived from single-center observational cohorts of postmenopausal women. Complete interventional clinical trials evaluating exosome-based therapies for metabolic bone diseases have not yet been reported. BMD, bone mineral density; BV/TV, bone volume/total volume; Tb.N, trabecular number; Tb.Sp, trabecular separation; AUC, area under the receiver-operating-characteristic curve.

**Table 3:** Comparative evaluation of major bone-targeted exosome engineering strategies across key performance and translation dimensions.

Strategy	Targeting Efficiency	Loading Capacity	Stability	Scalability	Immunogenicity	Off-Target Effects	Regulatory Feasibility
Membrane surface modification	High for bone matrix/mineral; RGD targets bone-cell integrins	Limited; surface decoration only, requires separate loading	Moderate; short peptides and aptamers susceptible to in vivo enzymatic degradation	High; chemically defined ligands are cheap and reproducible	Low	Moderate; RGD also binds integrins on other tissues	Favourable; small defined conjugates
Cargo loading—endogenous pre-loading	Not applicable alone; no added homing	Low; limited by cellular packaging and low yield	High; cargo stably embedded during biogenesis	Low; difficult to standardize mass production	Low; naturally derived	High without targeting ligand; broad biodistribution (liver/spleen)	Challenging; batch variability and QC
Cargo loading—exogenous post-loading	Not applicable alone; no added homing	High; flexible, controllable loading of diverse payloads	Moderate; membrane perturbation and cargo leakage (esp. electroporation)	Moderate–high; more standardized but method-dependent	Low–moderate; membrane perturbation may expose epitopes	High without targeting ligand; broad biodistribution	Moderate; needs parameter/encapsulation QC
Biomimetic modification	High; strong bone-selective hydroxyapatite affinity	Depends on combined cargo method	High; stable hydroxyapatite binding	Moderate; chemical conjugation reproducible, cell-derived sources harder to scale	Low; avoids chemical-conjugation immune responses	Low; bone-selective	Favourable–moderate; alendronate is clinically approved

## Data Availability

All relevant data analyzed in this review are included in the publicly available published literature.

## Abbreviations

The following abbreviations are used in this manuscript:

ABPS	Achyranthes bidentata polysaccharide
AKT	Protein kinase B
ALN	Alendronate
ALP	Alkaline phosphatase
BMSC	Bone marrow mesenchymal stem cell
BMP4	Bone morphogenetic protein 4
β-TCP	β-tricalcium phosphate
BMD	Bone mineral density
BV/TV	Bone volume/total volume
circRNA	Circular RNA
Col1	Type I collagen
EMs	Exosome mimetics
EVs	Extracellular vesicles
FAK	Focal adhesion kinase
HA	Hydroxyapatite
HDAC7	Histone deacetylase 7
hiPSC	Human induced pluripotent stem cell
IL-6	Interleukin-6
JNK	c-Jun N-terminal kinase
lncRNA	Long non-coding RNA
miRNA	MicroRNA
MISEV2018	Minimal information for studies of extracellular vesicles 2018
MSU	Monosodium urate
MVBs	Multivesicular bodies
NF-κB	Nuclear factor-kappa B
NFATc1	Nuclear factor of activated T-cells cytoplasmic 1
NLRP3	NOD-like receptor pyrin domain-containing protein 3
OCN	Osteocalcin
OPG	Osteoprotegerin
PI3K	Phosphatidylinositol 3-kinase
PTEN	Phosphatase and tensin homolog
PTH	Parathyroid hormone
PGE2	Prostaglandin E2
QC	Quality control
RANK	Receptor activator of nuclear factor-kappa B
RANKL	Receptor activator of nuclear factor-kappa B ligand
RGD	Arg-Gly-Asp peptide
Runx2	Runt-related transcription factor 2
SAG	Smoothened agonist
sEVs	Small extracellular vesicles
siRNA	Small interfering RNA
TET3	Ten-eleven translocation methylcytosine dioxygenase 3
TNF-α	Tumor necrosis factor-α
Wnt	Wingless-related integration site
